# A Large Real-World Study on the Effectiveness of the Combined Inhibition of EGFR and MET in EGFR-Mutant Non-Small-Cell Lung Cancer After Development of EGFR-TKI Resistance

**DOI:** 10.3389/fonc.2021.722039

**Published:** 2021-10-01

**Authors:** Li Liu, Jingjing Qu, Jianfu Heng, Chunhua Zhou, Yi Xiong, Haiyan Yang, Wenjuan Jiang, Liang Zeng, Songlin Zhu, Yongchang Zhang, Jiarong Tan, Chengping Hu, Pengbo Deng, Nong Yang

**Affiliations:** ^1^ Department of Lung Cancer and Gastroenterology, Hunan Cancer Hospital, Affiliated Tumor Hospital of Xiangya Medical School of Central South University, Changsha, China; ^2^ Department of Respiratory Disease, Thoracic Disease Centre, The First Affiliated Hospital, College of Medicine, Zhejiang University, Hangzhou, China; ^3^ Department of Clinical Pharmaceutical Research Institution, Hunan Cancer Hospital, Affiliated Tumor Hospital of Xiangya Medical School of Central South University, Changsha, China; ^4^ Department of Respiratory Medicine, Xiangya Hospital of Central South University, Changsha, China

**Keywords:** EGFR mutation, MET amplification, EGFR-TKI and crizotinib combination, NSCLC, EGFR-TKI resistance

## Abstract

**Background:**

*MET* proto-oncogene amplification (amp) is an important mechanism underlying acquired resistance to epidermal growth factor receptor (EGFR) tyrosine kinase inhibitors (TKIs). However, the optimal treatment strategy after acquiring *MET-*amp-mediated EGFR-TKI resistance remains controversial. Our study compared three treatment strategies for patients with *EGFR*-mutant non-small-cell lung cancer (NSCLC) who were detected with *MET-*amp at EGFR-TKI progression using next-generation sequencing.

**Methods:**

Of the 70 patients included in the study, 38 received EGFR-TKI + crizotinib, 10 received crizotinib monotherapy, and 22 received chemotherapy. Clinical outcomes and molecular profiles were analyzed.

**Results:**

The objective response rate was 48.6% for EGFR-TKI + crizotinib group, 40.0% for crizotinib monotherapy group, and 18.2% for chemotherapy group. Patients who received EGFR-TKI + crizotinib had significantly longer progression-free survival than those who received crizotinib or chemotherapy (5.0 *vs*. 2.3 *vs*. 2.9 months, p = 0.010), but overall survival was comparable (10.0 *vs*. 4.1 *vs*. 8.5 months, p = 0.088). *TP53* mutation (58.5%) and *EGFR-*amp (42.9%) were frequent concurrent mutations of the cohort. Progression-free survival was significantly longer for patients with either concurrent *TP53* mutation (n = 17) (6.0 *vs*. 2.3 *vs*. 2.9 months, p = 0.009) or *EGFR-*amp (n = 13) (5.0 *vs*. 1.2 *vs*. 2.4 months, p = 0.016) in the EGFR-TKI + crizotinib group than the other two regimen. Potential acquired resistance mechanisms to EGFR-TKI + crizotinib included *EGFR*-T790M (n = 2), *EGFR-*L718Q (n = 1), *EGFR*-S645C (n = 1), *MET*-D1228H (n = 1), *BRAF*-V600E (n = 1), *NRAS*-Q61H (n = 1), *KRAS-*amp (n = 1), *ERBB2-*amp (n = 1), *CDK4-*amp (n = 1), and *MYC-*amp (n = 1).

**Conclusion:**

Our study provides real-world clinical evidence from a large cohort that simultaneous inhibition of *EGFR* and *MET* could be a more effective therapeutic strategy for patients with *MET-*amp acquired from EGFR-TKI therapy.

## Introduction

The discovery of oncogenic drivers has transformed the management of advanced non-small-cell lung cancer (NSCLC) into a more personalized approach by tailoring the treatment regimen of a patient based on the presence or absence of molecular biomarkers (1). Sensitizing mutations in epidermal growth factor receptor (EGFR) are the most common predictive biomarkers and are identified in approximately 60% of Chinese patients with advanced NSCLC (2). In patients with *EGFR*-mutant advanced NSCLC, frontline therapy with EGFR tyrosine kinase inhibitors (TKIs) provides superior clinical outcomes as compared to chemotherapy (3–6). However, resistance to EGFR-TKI inevitably develops in most patients within 10–12 months of therapy through the acquisition of either EGFR-dependent or EGFR-independent mechanisms (7–10). *EGFR* T790M is the most frequent mechanism of acquired resistance to first- and second-generation EGFR-TKIs and is successfully targeted by third-generation EGFR-TKIs (11). EGFR-independent pathways that bypass EGFR inhibition, including aberrations in *MET* proto-oncogene (MET), also mediate EGFR-TKI resistance (7–10). MET, a receptor tyrosine kinase, is activated by the binding of hepatocyte growth factor (HGF), leading to the activation of various downstream signaling pathways including the phosphatidylinositol-4-biphosphate 3-kinase (PI3K) pathway (12–14). Dysregulation of MET signaling is implicated as one of the oncogenic drivers in lung cancer development and progression (12–14). *MET* gene amplification has been reported in 2–8% of EGFR-TKI-naive advanced NSCLCs and in approximately 20% of EGFR-TKI-treated relapsed NSCLCs (8, 9, 15, 16). Moreover, patients who harbor concurrent *EGFR* mutation with *MET* amplification after EGFR-TKI progression demonstrated short progression-free and overall survival outcomes with MET-TKI monotherapy (16), indicating the urgent need for identifying effective therapeutic strategies that could overcome the *MET* amplification-mediated EGFR-TKI resistance.

The reciprocal crosstalk between EGFR and MET in lung adenocarcinoma suggests that simultaneous inhibition through the combined use of EGFR-TKI and MET-TKI would benefit and potentially improve the survival outcomes of patients with concurrent *EGFR* and *MET* aberrations (17, 18). Over the years, a growing number of studies have demonstrated the clinical benefit of different combinations of various generations of EGFR-TKIs and various MET-TKIs including crizotinib (16, 19–23), capmatinib (21, 24), and savolitinib (25–27). Recently, tepotinib plus gefitinib showed promising antitumor activity in NSCLC with concomitant *EGFR* mutation and *MET* amplification or high MET overexpression compared with chemotherapy (28). The promising clinical outcomes demonstrated by the combinatorial therapy of EGFR-TKI and MET-TKI highlight the survival benefit of targeted therapy over chemotherapy or possibly MET-TKI monotherapy. However, due to the lack of solid clinical evidence derived from a larger cohort or a controlled clinical trial, there is still no consensus on the standard treatment for patients with advanced *EGFR*-mutant NSCLC who acquire *MET* amplification during EGFR-TKI therapy. In current clinical practice, after progression from EGFR-TKI with *MET* amplification or MET overexpression, patients are managed with any of the three regimens: combination therapy consisting of an EGFR-TKI and a MET-TKI, MET-TKI monotherapy, or chemotherapy. In this real-world observational study, we aimed to identify the treatment regimen that could impart better clinical benefit for patients with concurrent *MET* amplification and *EGFR* sensitizing mutation after progression from EGFR-TKI therapy. To achieve this aim, we retrospectively analyzed and compared the clinical outcomes of 70 patients who were detected with *MET* amplification using next-generation sequencing (NGS) after progression from EGFR-TKI regimen and received any of the three regimens. We also explored the molecular mechanism of acquired resistance to the EGFR-TKI and crizotinib combination therapy.

## Patients and Methods

### Study Subjects and Inclusion/Exclusion Criteria

Patients diagnosed with advanced *EGFR*-mutant NSCLC who received treatment from Hunan Cancer Hospital or Xiangya Hospital between March 2015 and March 2020 were included in this study. The baseline *EGFR* mutation status was assessed from blood samples or tissue samples obtained by needle biopsy of lung lesions or lymph nodes using either NGS or amplification refractory mutation system (ARMS) PCR. At progression from EGFR-TKI therapy, all patients underwent rebiopsy of tissue, blood, or other biological samples for NGS analysis. Patients with confirmed mutation status for *EGFR* and *MET* amplification at progression from first- or subsequent-line EGFR-TKI therapy were included in the study. Patients with baseline *MET* amplification or those who had received prior crizotinib therapy were excluded from the study. The patient data were retrieved from the medical records. This study was approved by the Institutional Ethics Committee of Hunan Cancer Hospital. All the patients provided written informed consent for the use of their data for research purposes.

### Treatment Procedures

Crizotinib at 250 mg twice daily was administered with or without continued treatment of EGFR-TKI. Patients without *EGFR* T790M were administered with erlotinib, gefitinib, or afatinib daily at an oral dose of 150, 250, or 40 mg, respectively. Patients with *EGFR* T790M received osimertinib at a daily oral dose of 80 mg. Some patients were administered platinum-based chemotherapy combined with pemetrexed (500 mg/cm^2^, 21 days per cycle). Treatment was continued until one or more of the following conditions occurred: unacceptable toxicity, disease progression or death, patient refusal, or treatment withdrawal for any other reason including pregnancy. Treatment responses were investigator assessed every two cycles (approximately 2 months) using enhanced computed tomography (CT) scanning according to the Response Evaluation Criteria in Solid Tumors (RECIST) version 1.1 (29). Objective response rate (ORR) was defined as the proportion of patients achieving complete response (CR) or partial response (PR). Disease control rate (DCR) was defined as the proportion of patients achieving CR, PR, and stable disease (SD). Treatment-related toxicity was evaluated according to the Common Terminology Criteria for Adverse Event (CTCAE) version 4.03. Real-world progression-free survival (rwPFS) was defined as the period between the date of initiating the treatment regimen with crizotinib, EGFR-TKI combined with crizotinib, or pemetrexed combined with platinum and the date of treatment discontinuation due to radiologically confirmed disease progression or intolerable side effect or death. Overall survival (OS) was defined as the period between the date of treatment administration after developing *MET* amplification-mediated EGFR-TKI resistance until death or the day of the last follow-up.

### Preparation of Circulating Cell-Free DNA and Tissue DNA

Circulating cell-Free DNA (cfDNA) was extracted from plasma samples and other liquid biopsy samples according to the manufacturer’s instructions using a QIAamp Circulating Nucleic Acid Kit (Qiagen, Hilden, Germany). Tissue DNA was extracted from formalin-fixed paraffin-embedded (FFPE) cell blocks of tumor biopsy or other cytology samples using QIAamp DNA FFPE tissue kit (Qiagen, Hilden, Germany). CfDNA and tissue DNA concentration were quantified using Qubit 2.0 fluorometer with dsDNA HS Assay Kit (Life Technologies, CA, USA).

### NGS-Based Analysis of the MET and EGFR Mutation Status

NGS was performed to establish the mutation status of *EGFR* and *MET* and to elucidate the molecular mechanism of acquired resistance to EGFR-TKI and crizotinib. NGS library construction, sequencing procedures, and sequencing data analysis were performed using optimized protocols at Burning Rock Biotech, a clinical laboratory accredited and certified by the College of American Pathologists (CAP) and Clinical Laboratory Improvement Amendments (CLIA), respectively (30). Target capture was performed using commercial gene panels consisting of 8–168 cancer-related genes. All gene panels interrogated all exons and critical introns of the classic lung cancer oncogenic genes including *EGFR*, *ALK*, *ROS*, and *MET*. The quality and size of the fragments were assessed using Agilent high-sensitivity DNA assay kit with the Bioanalyzer 2100 (Agilent, CA, USA). Sequencing of the indexed samples was performed using a NextSeq500 instrument (Illumina, CA, USA) with paired-end reads at a target sequencing depth of 10,000× for plasma samples and 1,000× for tissue samples.

DNA sequence data were analyzed using an optimized bioinformatics pipeline developed by Burning Rock Biotech, which accurately detected various cancer-related mutation types, including single nucleotide, copy number, and structural variations (30). Sequence mapping to the reference human genome (hg19) was performed using Burrows–Wheeler Aligner (version 0.7.10). Genomic analysis toolkit (GATK version 3.2) and VarScan (version 2.4.3) were used for local alignment optimization, variant calling, and annotation. Plasma samples were compared against paired white blood cells to filter out clonal hematopoiesis-related variants and identify true somatic variants. Factera (version 1.4.3) was used to analyze structural variants. Copy number variations (CNVs) were analyzed based on the depth of coverage and were called when the coverage data of the gene region was quantitatively and statistically significant from its reference control. The copy number cutoff for identifying CN deletion was set at 1.5 and CN amplification set at 2.25.

### Statistical Analysis

Mann–Whitney U test was implemented for intergroup comparisons of continuous variables. Fisher’s exact test was used to compare the ORR and DCR among the groups. Survival analyses were performed for each group using the Kaplan–Meier method with log-rank statistics. All the statistical analysis was performed using R statistics package (R version 3.5.3; Vienna, Austria). Statistical significance was defined as p < 0.05. p-values (adjP) were adjusted accordingly with clinical factors including age, smoking history, and treatment line. Hazard ratios (HRs) and their corresponding 95% confidence intervals (CIs) were also calculated using Cox proportional hazards regression method for adjusted survival curves.

## Results

### Patient Characteristics

**Figure 1:**
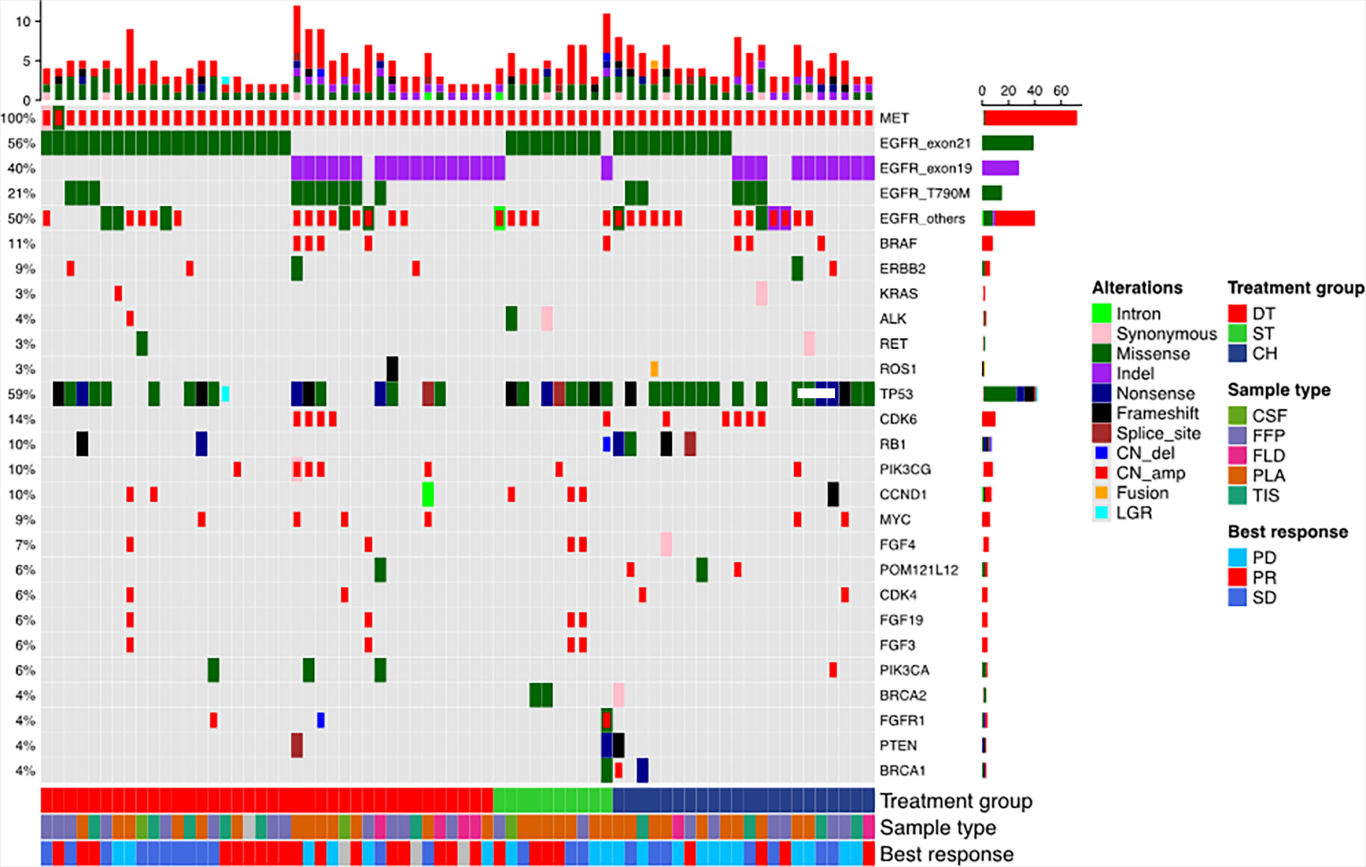
Mutation landscape of the cohort before receiving the three regimens: EGFR-TKI combined with crizotinib (DT), crizotinib monotherapy (ST), and chemotherapy (CH). The patient’s best responses to the treatment are also annotated at the bottom of the oncomap.

Seventy patients who were detected with *MET* amplification using DNA NGS after progression from EGFR-TKI therapy were included in this study. *MET* amplification was detected after progression from first-generation EGFR-TKI therapy in 57.1% (n = 40) of patients, after progression from second-generation EGFR-TKI therapy in 4.3% (n = 3), and after progression from third-generation EGFR-TKI therapy in 37.1% (n = 26) of patients. *MET* amplification was not evaluated using fluorescence *in situ* hybridization due to lack of or insufficient tissue biopsy sample. The cohort was further stratified into three groups based on the treatment they received after EGFR-TKI progression: 38 patients received a combination of crizotinib and any EGFR-TKI, 10 patients received crizotinib monotherapy, and 22 patients received carboplatin and pemetrexed chemotherapy. All the patients had extensive metastasis at EGFR-TKI progression. Most of the patients included in this study progressed from first-line EGFR-TKI and received one of the three regimens described above as second-line therapy (57.9% *vs*. 70.0% and 68.2%, p = 0.001). Of the 38 patients who received EGFR-TKI plus crizotinib therapy, 13 patients received first-generation EGFR-TKI (i.e., gefitinib, erlotinib), 1 patient received second-generation EGFR-TKI (i.e., afatinib), and 24 patients received third-generation EGFR-TKI (i.e., osimertinib). The median age was 56 years (range, 48–65) for the patients who received EGFR-TKI plus crizotinib therapy, 57 years (range, 55–68) for patients who received crizotinib monotherapy, and 56 years (range, 50–63) for patients who received chemotherapy. The therapeutic regimens were independent of sex, age, smoking status, and mutation detection method. The concurrent *EGFR* mutations of the cohort were as follows: 55.7% (39/70) *EGFR* exon 21 L858R mutation, 40.0% (28/70) *EGFR* exon 19 deletion (19del), 21.4% (15/70) *EGFR* T790M mutation with either L858R (n = 5) or 19del (n = 10), and 50.0% (35/70) other *EGFR* mutations (**Figure 1**). **Table 1** summarizes the clinicopathological characteristics of the cohort. The treatment groups had comparable clinical features including age, sex, smoking history, Eastern Cooperative Oncology Group performance status (ECOG PS), and prior EGFR-TKI therapy. However, when two groups were compared with each other, ECOG PS remained comparable between the EGFR-TKI plus crizotinib combination therapy group and crizotinib monotherapy group (p = 1) but was significantly different between EGFR-TKI plus crizotinib combination therapy group and chemotherapy group (p = 0.023) and crizotinib monotherapy group and chemotherapy group (p = 0.024).

**Table 1:** Baseline demographic and treatment characteristics of patients with concurrent *EGFR* mutations and *MET* amplification after EGFR-TKI resistance.

Clinical features	n (%)				
	Overall (n = 70)	EGFR-TKI+crizotinib (n = 38)	Crizotinib (n = 10)	Chemotherapy (n = 22)	P
Age (median [range]) years	56 [50–65]	56 [48–65]	57 [55–68]	56 [50–63]	0.491
Sex					0.353
Female	40 (57.1)	23 (60.5)	7 (70.0)	10 (45.5)	
Male	30 (42.9)	15 (39.5)	3 (30.0)	12 (54.5)	
Smoking history					0.625
Never smoker	47 (67.1)	25 (65.8)	7 (70.0)	15 (68.2)	
Current/former smoker	17 (24.3)	8 (21.1)	3 (30.0)	6 (27.3)	
No data	6 (8.6)	5 (13.2)	0 (0.0)	1 (4.5)	
ECOG PS score before receiving the study regimens					
0	9 (12.9)	7 (18.4)	2 (20.0)	0 (0)	0.115
1	57 (81.4)	28 (73.7)	7 (70.0)	22 (100)	
2	4 (5.7)	3 (7.9)	1 (10.0)	0 (0)	
>2	0 (0)	0 (0)	0 (0)	0 (0)	
Treatment line for study regimens					0.001
2	44 (62.9)	22 (57.9)	7 (70.0)	15 (68.2)	
3	23 (32.9)	16 (42.1)	0 (0.0)	7 (31.8)	
4	2 (2.9)	0 (0.0)	2 (20.0)	0 (0.0)	
7	1 (1.4)	0 (0.0)	1 (10.0)	0 (0.0)	
Treatment regimen received after progression from EGFR-TKI (study regimens)					<0.001
1^st^ EGFR-TKI+crizotinib	13 (18.6)	13 (34.2)	0 (0.0)	0 (0.0)	
2^nd^ EGFR-TKI+crizotinib	1 (1.4)	1 (2.6)	0 (0.0)	0 (0.0)	
3^rd^ EGFR-TKI+crizotinib	24 (34.3)	24 (63.2)	0 (0.0)	0 (0.0)	
Crizotinib monotherapy	10 (14.3)	0 (0.0)	10 (100.0)	0 (0.0)	
Chemotherapy	22 (31.4)	0 (0.0)	0 (0.0)	22 (100.0)	
Prior-line EGFR-TKI regimen received					0.238
1^st^ EGFR-TKI	40 (57.1)	20 (52.6)	7 (70.0)	13 (59.1)	
2^nd^ EGFR-TKI	3 (4.3)	2 (5.3)	0 (0.0)	1 (4.5)	
3^rd^ EGFR-TKI	26 (37.1)	16 (42.1)	2 (20.0)	8 (36.4)	
1st EGFR-TKI (after 2 lines of chemotherapy)	1 (1.4)	0 (0.0)	1 (10.0)	0 (0.0)	

ECOG PS, Eastern Cooperative Oncology Group performance score; EGFR-TKI, epidermal growth factor receptor tyrosine kinase inhibitor; 1st, first generation; 2nd, second generation; 3rd, third generation.

### Clinical Outcomes of Patients With Concurrent EGFR Mutation and MET Amplification

**Figure 2:**
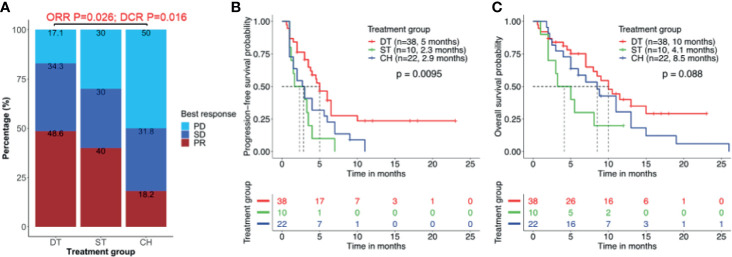
Clinical outcomes are better with combined therapy of EGFR-TKI and crizotinib. **(A)** Overall response rate (ORR) and disease control rate (DCR) were significantly higher in patients who received EGFR-TKI with crizotinib combination therapy (DT) than crizotinib monotherapy (ST) or chemotherapy (CH). **(B, C)** Combination therapy results in significantly longer median real-world progression-free survival (PFS) **(B)** but no statistically significant benefit in real-world overall survival **(C)**. Tick marks denote censored patients.

Among the 38 patients who received EGFR-TKI plus crizotinib combination therapy, only 35 patients were evaluable for best response. Of them, 48.6% (17/35) achieved PR and 34.3% (12/35) achieved SD, resulting in an ORR of 48.6% and DCR of 82.9%. Only 17.1% (6/35) did not benefit from the EGFR-TKI plus crizotinib. Of the 10 patients who received crizotinib monotherapy, 40.0% (4/10) and 30.0% (3/10) each achieved PR and SD, and 30% (3/10) had no clinical benefit, resulting in an ORR of 40.0% and a DCR of 70.0%. Of the 22 patients who received chemotherapy regimen, 18.2% (4/22) achieved PR, 31.8% (7/22) achieved SD, and 50.0% (11/22) had no clinical benefit, resulting in an ORR of 18.2% and a DCR of 50.0%. As compared to patients who received chemotherapy, patients who received EGFR-TKI plus crizotinib had significantly better ORR (p = 0.026) and DCR (p = 0.016) but was not statistically different from that of the patients who received crizotinib monotherapy (**Figure 2A**).

Patients who received EGFR-TKI plus crizotinib had significantly longer median rwPFS as compared to those who received crizotinib monotherapy [5.0 *vs*. 2.3 months; p = 0.007; HR = 0.88, 95% CI (0.42, 2.42), adjp = 0.036] and chemotherapy regimen (5.0 *vs*. 2.9 months; p = 0.038; HR = 0.72, 95% CI (0.32, 2.06), adjp = 0.024] (**Figure 2B**). However, patients who received EGFR-TKI plus crizotinib had a trend of longer OS, with a median OS of 10.0 months but did not reach statistical significance [*vs*. crizotinib p = 0.058; HR = 0.65, 95% CI (0.48, 1.92), adjp = 0.17; *vs*. chemotherapy p = 0.095; HR = 0.59, 95% CI (0.35, 1.8), adjp = 0.20; **Figure 2C**). Median OS was 4.1 months for those who received crizotinib monotherapy and 8.5 months for those who received chemotherapy. The ORR (p = 0.22), DCR (p = 0.45), rwPFS (p = 0.30, adjp = 0.96), and OS (p = 0.21, adjp = 0.66) were comparable between patients who received crizotinib monotherapy and chemotherapy.

### Molecular Factors Affecting Survival Outcomes

**Figure 3:**
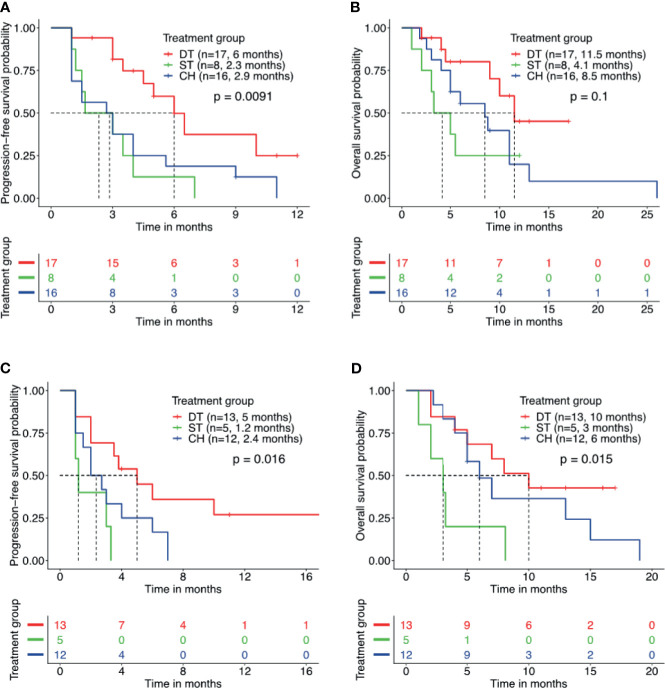
Patients with concurrent *TP53* mutation and *EGFR* amplification benefit from combined therapy of EGFR-TKI and crizotinib. Kaplan–Meier curves for progression-free survival **(A, C)** and overall survival **(B, D)** demonstrate the survival benefit of patients with concurrent *TP53* mutation and *EGFR* amplification who received combined therapy of EGFR-TKI and crizotinib (DT) than crizotinib monotherapy (ST) or chemotherapy (CH). Tick marks denote censored patients.

Furthermore, we studied the impact of other molecular factors on the survival outcome from the three regimens. Numerous studies have implicated the detection of concurrent *TP53* mutations in poorer survival outcomes with EGFR-TKI and crizotinib therapy (31–35). Concurrent *TP53* mutation was detected in 58.5% of our cohort and the most frequent co-occurring mutation. *TP53* mutation was detected in 44.7% (17/38) of patients who received EGFR-TKI plus crizotinib, 80.0% (8/10) of patients who received crizotinib monotherapy, and 72.7% (16/22) of patients who received chemotherapy. Among the patients with concurrent *TP53* mutation, those who received EGFR-TKI plus crizotinib (n = 17) had a significantly longer rwPFS than those who received crizotinib monotherapy (n = 8) (6.0 *vs*. 2.3 months, p = 0.007, adjp = 0.208) or chemotherapy (n = 16) (6.0 *vs*. 2.9 months, p = 0.016, adjp = 0.048) (**Figure 3A**). Patients with concurrent *TP53* mutation who received EGFR-TKI plus crizotinib (n = 17) had a trend of longer OS but statistically comparable with the patients who received crizotinib monotherapy (n = 8) (11.5 *vs*. 4.1 months, p = 0.077, adjp = 0.599) or chemotherapy (n = 16) (11.5 *vs*. 8.5 months, p = 0.074, adjp = 0.162) (**Figure 3B**). Median rwPFS (p = 0.55, adjp = 0.85) and OS (p = 0.49, adjp = 0.99) were comparable for patients with concurrent *TP53* mutations who received crizotinib monotherapy and chemotherapy.

In addition to concurrent *TP53* mutation status, the prognostic effect of *EGFR* amplification was also investigated. Concurrent *EGFR* amplification was detected in 42.9% (30/70) of the cohort and the second most frequently co-occurring mutation. Among the patients with concurrent *EGFR* amplification, those who received EGFR-TKI plus crizotinib (n = 13) had a significantly longer rwPFS (5.0 *vs*. 1.2 months, p = 0.017, adjp = 0.046) and OS (10.0 *vs*. 3.0 months, p = 0.02, adjp = 0.019) than those who received crizotinib monotherapy (n = 5) (**Figures 3C, D**). Meanwhile, patients with concurrent *EGFR* amplification who received EGFR-TKI plus crizotinib (n = 13) had no statistical difference in rwPFS (5.0 *vs*. 2.4 months, p = 0.06, adjp = 0.094) and OS (10.0 *vs*. 6.0 months, p = 0.35, adjp = 0.44) than patients who received chemotherapy (n = 12) (**Figures 3C, D**). Taken together, these data indicate that concurrent *TP53* mutations and *EGFR* amplifications that are known to affect the prognosis of patients with *EGFR*-mutant NSCLC did not affect the survival outcomes of EGFR-TKI plus crizotinib combination therapy.

### Exploring the Underlying Acquired Resistance Mechanism in the EGFR-TKI Plus Crizotinib Cohort

**Figure 4:**
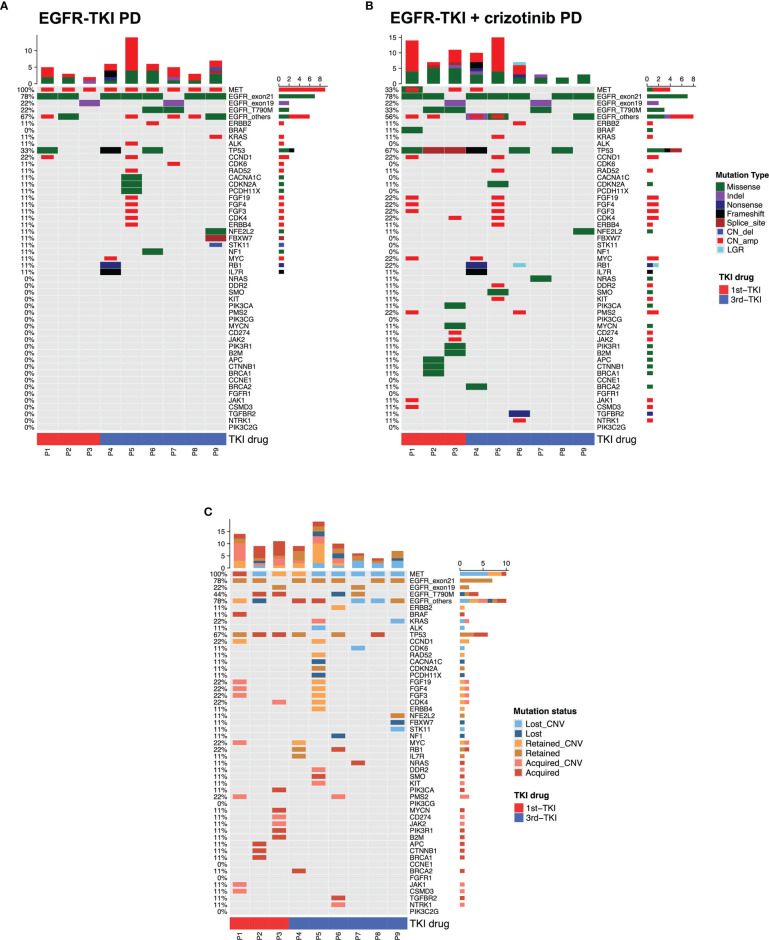
Mutation landscape of the nine evaluable patients after progression from EGFR-TKI with crizotinib. The oncomaps summarize the mutation profile that indicates the specific mutation types of the nine patients before receiving the combination therapy (at progression from EGFR-TKI) **(A)** and at progression from the combination therapy **(B)**. **(C)** This oncomap integrates the mutation profile before receiving the combination therapy and at progression to indicate the mutations retained, acquired (undetected at baseline), and lost (undetected at progression).

We further analyzed the molecular mechanism of acquired resistance developed during EGFR-TKI plus crizotinib by comparing the mutation profile before receiving the combination therapy and after disease progression. Among the 38 patients who received EGFR-TKI plus crizotinib, 9 patients (23.7%) submitted rebiopsy samples for NGS-based genomic testing after progression from the combination therapy. **Figure 4** illustrates the mutation profile at baseline (**Figure 4A**), at progression (**Figure 4B**), and the overlap of mutation profiles at baseline and progression to highlight the mutations retained, lost, or acquired at progression (**Figure 4C**). **Table 2** summarizes the molecular mechanisms of acquired resistance and the detailed clinical information of the nine evaluable patients. After progression, *MET* amplification was undetected in 66.7% (6/9) of the patients. EGFR-dependent acquired resistance mechanisms detected from the cohort included *EGFR* T790M (n = 2), *EGFR* L718Q (n = 1), and *EGFR* S645C (n = 1). Secondary mutation *MET* D1228H was detected in a patient. EGFR/MET-independent bypass mechanisms detected from our cohort included *BRAF* V600E (n = 1), *NRAS* Q61H (n = 1), *KRAS* amplification (n = 1), *ERBB2* amplification (n = 1), *CDK4* amplification (n = 1), and *MYC* amplification (n = 1). The two patients with unknown mechanisms of resistance had undetected *MET* amplification.

**Table 2:** Detailed treatment regimen and molecular mechanisms of acquired resistance to EGFR-TKI and crizotinib combination therapy of the 9 evaluable patients.

Patient number	Baseline *EGFR* sensitizing mutation	Previous EGFR-TKI regimen received	*MET* amplification copy number	Regimen received	Best response (% change in tumor size)/progression-free survival	*MET* amplification status at progression	Potential molecular mechanisms detected at progression
P01	L858R	Gefitinib	5.6	Gefitinib + crizotinib	SD (−24%)/10.0 months	Retained (CN = 6.6)	*BRAF* V600E + *MET* D1228H + *MYC* amplification
P02	L858R	Erlotinib	3.55	Erlotinib + crizotinib	SD (−10%)/6.5 months	Lost	*EGFR* T790M
P03	Exon 19 deletion	Erlotinib	21.24	Erlotinib + crizotinib	PR (−62%)/6.5 months	Retained (CN = 3.62)	*EGFR* T790M + *CDK4* amplification
P04	L858R	1L Erlotinib; 2L Osimertinib	2.4	Osimertinib + crizotinib	98SD (−5%)/5.0 months	Retained (CN = 3.5)	*EGFR* S645C
P05	L858R	Erlotinib	4.98	Osimertinib + crizotinib	PD (+22%)/2.0 months	Lost	*EGFR* L718Q + *KRAS* amplification
P06	L858R + T790M	1L Erlotinib; 2L Osimertinib	6.39	Osimertinib + crizotinib	SD (0%)/3.0 months	Lost	*ERBB2* amplification
P07	exon 19 deletion+T790M	1L Erlotinib; 2L Osimertinib	4.91	Osimertinib + crizotinib	PD (+56%)/1.0 month	Lost	*NRAS* Q61H
P08	L858R	1L Erlotinib; 2L Osimertinib	2.88	Osimertinib + crizotinib	SD (−18%)/6.0 months	Lost	Unknown
P09	L858R	1L Gefitinib; 2L Osimertinib	2.5	Osimertinib + crizotinib	PD (target −52%; new liver met)/1.5 months	Lost	Unknown

CN, copy number; 1L, first-line; 2L, second-line; PD, progressive disease; PR, partial response; SD, stable disease.

### Safety and Toxicity Profiles

**Table 3** summarizes the treatment-related toxicities. The most frequently reported treatment-related toxicities were neutropenia (n = 16, 22.9%) and fatigue (n = 13, 18.6%). Of the 38 patients who received EGFR-TKI combined with crizotinib, grade 1–2 neutropenia (n = 5), grade 1–3 elevated transaminase (n = 4), grade 1–2 vomiting (n = 4), grade 1–2 diarrhea (n = 4), and grade 1–3 rash (n = 4) were the most frequent toxicity. Among the 10 patients who received crizotinib monotherapy, the most frequent toxicities were grade 1–2 vomiting (n = 3) and diarrhea (n = 2). Of the 22 patients who received chemotherapy regimen, grade 1–3 neutropenia (n = 10) and fatigue (n = 8) were the most frequent toxicity. Grade 3 or above treatment-related toxicities were observed in six patients (15.8%) who received EGFR-TKI plus crizotinib and three patients (13.6%) who received chemotherapy (p = 0.591). No grade IV, fatal, or unexpected adverse events were observed in our cohort.

**Table 3:** Adverse events in each treatment group.

Adverse events	EGFR-TKI + crizotinib (n = 38)			Crizotinib (n = 10)			Chemotherapy (n = 22)		
	Grade 1-2	Grade ≥3	Total	Grade 1-2	Grade ≥3	Total	Grade 1-2	Grade ≥3	Total
Neutropenia	5 (13.2%)	0	5 (13.2%)	1 (10.0%)	0	1 (10.0%)	9 (40.9%)	1 (4.5%)	10 (45.5%)
Fatigue	2 (5.3%)	2 (5.3%)	4 (10.5%)	1 (10.0%)	0	1 (10.0%)	6 (27.2%)	2 (9.1%)	8 (36.4%)
Elevated transaminase	3 (7.9%)	1 (2.6%)	4 (10.5%)	1 (10.0%)	0	1 (10.0%)	4 (18.2%)	0	4 (18.2%)
Vomiting	4 (10.5%)	0	4 (10.5%)	3 (30.0%)	0	3 (30.0%)	2 (9.1%)	0	2 (9.1%)
Diarrhea	4 (10.5%)	0	4 (10.5%)	2 (20.0%)	0	2 (20.0%)	1 (4.5%)	0	1 (4.5%)
Rash	3 (7.9%)	1 (2.6%)	4 (10.5%)	0	0	0	0	0	0
Onychia	2 (5.3%)	1(2.6%)	3 (7.9%)	0	0	0	0	0	0
Myalgia	0	0	0	0	0	0	1 (4.5%)	0	1 (4.5%)
Interstitial pneumonia	0	1 (2.6%)	1 (2.6%)	0	0	0	0	0	0

### Case Vignette

A 45-year old man with 60 pack-year smoking history (P05, **Table 2**) was diagnosed with *EGFR* L858R-mutant stage IV lung adenocarcinoma and received a combination of osimertinib and crizotinib after acquiring *MET* amplification with an absolute CN of 4.98 at disease progression from first-line erlotinib therapy. The combination therapy was well tolerated except for grade 2 vomiting. It is interesting that his primary lung lesion achieved a dramatic response within 4 weeks of the combination therapy and remained responsive for 3 months (chest CT images, **Supplementary Figure S1**) despite the development of new metastatic lesions in the liver (abdominal CT images, **Supplementary Figure S1**), which led to rapid disease progression and eventual demise on January 2018. At progression, NGS analysis of the liver lesions identified *EGFR* L718Q, *KRAS* CN amplification, and the absence of *MET* amplification, suggesting that inhibitor resistance in the liver lesions might be mediated by both EGFR-dependent and EGFR-independent mechanisms (**Supplementary Figure S1**).

## Discussion

The clinical success of EGFR-TKI is consistently limited by the acquisition of resistance, which inevitably occurs in almost all patients within 1 year of EGFR-TKI therapy. *MET* amplification-mediated resistance to EGFR-TKI occurs in approximately 20% of patients who progress on various generations of EGFR-TKI (8, 9, 15). Until now, there is still no consensus on the optimal subsequent-line treatment regimen for patients who acquired *MET* amplification-mediated EGFR-TKI resistance. The efficacy of crizotinib either alone or in combination with any EGFR-TKI has been explored among patients who were detected with *MET* amplification after progression from EGFR-TKI treatment (16, 19, 20, 22, 36, 37). Due to the limited sample size, the data in these studies were inadequate to strongly support the standard clinical use of combination therapies targeting both *EGFR* and *MET* after *MET* amplification-mediated EGFR-TKI resistance. To shed light on which treatment regimen is the most effective in this subset of patients, we retrospectively analyzed the clinical outcomes of 70 patients with *EGFR*-mutant NSCLC who acquired *MET* amplification during EGFR-TKI therapy and received any of the following three treatment regimens: crizotinib in combination with any EGFR-TKI (n = 38), crizotinib monotherapy (n = 10), or chemotherapy regimen (n = 22). Our study revealed a significantly higher real-world ORR, DCR, and PFS and a trend of longer OS for patients who received the combination therapy of EGFR-TKI and crizotinib than those who received either crizotinib monotherapy or chemotherapy regimen, providing clinical evidence that the combined inhibition of *EGFR* and *MET* provides clinical benefit to these patients after EGFR-TKI progression. The superior clinical outcome for the combination therapy and the similar clinical outcomes for crizotinib monotherapy and chemotherapy suggested four important points. First, NGS-based analysis of samples obtained after EGFR-TKI progression is instrumental in identifying concurrent actionable mutations to improve clinical outcomes. Second, *MET* amplification status identified by NGS could potentially serve as a predictive biomarker for response to crizotinib therapy after EGFR-TKI progression. Third, despite developing EGFR-TKI resistance, the tumor/s of patients detected with concurrent *EGFR* mutation and *MET* amplification at disease progression have acquired “addiction” to MET but remains “addicted” to EGFR. Fourth, the continued use of EGFR-TKI is necessary to target the remaining *EGFR*-addicted clones to improve clinical outcomes. To the best of our knowledge, our study is the first to include the largest cohort to compare the three subsequent-line treatment regimens after EGFR-TKI progression in the real-world setting.

Despite the clinical benefit of EGFR-TKI combined with crizotinib, these clinical responses appeared to be non-durable and heterogeneous. We speculate that the differences in *MET* copy number and inherent genetic heterogeneity of tumors might underlie the heterogeneous and short-lived clinical outcomes observed in our cohort. Consistent with our findings, another study reported the heterogeneous responses in patients with marked shrinkage of the primary lesion but continued progression of other metastatic lesions (20). *MET* amplification may also not be mutually exclusive from other molecular pathways that mediate EGFR-TKI resistance and could contribute to short-lived and heterogeneous responses of patients who receive MET-TKI after progression from EGFR-TKI therapy (20).

In addition to crizotinib, the potential effectiveness of more selective MET-TKIs, including capmatinib, savolitinib, and tepotinib, combined with any EGFR-TKIs in the management of patients with *EGFR-*mutant, *MET*-amplified NSCLC has been demonstrated in several prospective clinical studies (21, 24–26, 28). Capmatinib combined with gefitinib was shown in a phase Ib/II study to be effective in patients with *EGFR*-mutated, *MET*-amplified/overexpressed NSCLC after progressing from prior EGFR-TKI therapy, with an ORR of 47% among patients with fluorescence *in situ* hybridization-based *MET* CN of ≥6 (24). Moreover, osimertinib and savolitinib combination therapy showed promising results for patients with *MET*-driven resistance to either first- or second-generation EGFR-TKIs without T790M, with an ORR of 64% and a median PFS of 9.1 months (25, 26). Moreover, the efficacy of savolitinib in combination with either gefitinib (31% PR, 16/51) or osimertinib (40% PR, 19/47) was demonstrated in patients with *EGFR*-mutant NSCLC with concurrent *MET* amplification after progression on first-line EGFR-TKI treatment (27). Among the patients with *MET*-amplified NSCLC included in the INSIGHT study, ORR was higher among those who received tepotinib plus gefitinib than chemotherapy [66.7% (12/18) *vs* 42.9% (3/7)] (28). With the growing number of clinical evidence on the superiority of EGFR-TKI and MET-TKI combination therapy over chemotherapy, we anticipate the consensus on the use of the combination therapy as the standard of care for the management of NSCLC after *MET* amplification-mediated resistance to EGFR-TKI.

Although mechanisms of acquired resistance to EGFR-TKI monotherapy and MET-TKI monotherapy have been well-elucidated (9, 10, 21, 26, 38–44), the mechanism mediating resistance to the combination therapy of EGFR-TKI and MET-TKI remains unclear. As expected, acquired mutations observed in our cohort were on- and off-target resistance mechanisms previously reported for monotherapy of EGFR-TKI and crizotinib. Acquisition of missense mutations in *MET* D1228, including D1228H, was implicated as one of the on-target resistance mechanisms to crizotinib therapy that can potentially be reversed by next-generation MET-TKIs (45). An *in vitro* study demonstrated that *EGFR* S645C can promote tumor formation and is less sensitive to erlotinib (39). *EGFR* L718Q has been reported as one of the rare resistance mechanisms to osimertinib therapy in an *EGFR* L858R/T790M-positive lung adenocarcinoma patient, which might be sensitive to afatinib (46, 47). EGFR-independent resistance mechanisms, including copy number amplifications in *ERBB2*, genes involved in cell cycle regulation such as *CDK4*, and mutations in genes involved in MAPK/PIK3 signaling, such as *BRAF*, *KRAS*, and *NRAS*, have been reported (41). A study identified *NRAS* Q61K as an acquired resistance mechanism to erlotinib in *EGFR* L858R-mutant cell line (38). *MYC* amplification was implicated in primary crizotinib resistance in *ALK*-rearranged NSCLC (44). *KRAS* amplification was implicated as the mechanism of resistance from crizotinib treatment in *MET* exon 14 mutant-NSCLC in a study using patient tumor-derived cells and xenografts, which was effectively targeted by dual MET/PI3K inhibition (42). Nonetheless, these acquired resistance mechanisms suggest that rebiopsy at progression is necessary to identify potentially actionable mutations for therapeutic decisions. These results also indicate that current therapeutic strategies for reversing acquired resistance to EGFR-TKI or crizotinib are applicable after progression from the EGFR-TKI and crizotinib combination therapy.

Our study has several limitations. First, it is a retrospective, non-randomized study conducted in two institutions from the same city, which could result in patient selection bias. Second, *MET* amplification status of our cohort was only determined using DNA NGS and lacked data on standard *MET* amplification or overexpression status assessed using fluorescence *in situ* hybridization or immunohistochemistry. A multicenter collaboration is required to further establish our findings.

In conclusion, our findings provide real-world clinical evidence that the simultaneous inhibition of *EGFR* and *MET* by the combined use of EGFR-TKI and crizotinib is generally tolerable and provides benefit to patients with *EGFR*-mutant NSCLC who acquired *MET* amplification-mediated EGFR-TKI resistance. Our findings add to the accumulating evidence of the potential of combination therapy with EGFR-TKIs and MET-TKIs as the optimal subsequent-line therapeutic strategy for this subset of patients.

## Data Availability Statement

The datasets presented in this study can be found in online repository. The names of the repository and accession number can be found below: National Omics Data Encyclopedia (NODE), accession number OEP002682. The dataset can be accessed through the URL: http://www.biosino.org/node/project/detail/OEP002682.

## Ethics Statement

The studies involving human participants were reviewed and approved by Institutional Ethics Committee of Hunan Cancer Hospital (approval number: 2021-19). The patients/participants provided their written informed consent to participate in this study.

## Author Contributions

LL, JQ, PD, and NY conceived and designed the study. HY, CZ, WJ, CH, and LL provided patient information. JT and SZ collected the data. LL and JQ analyzed the data. PD and NY supervised the project and acquired financial support. All the authors contributed to manuscript writing, editing, and approving the manuscript.

## Funding

This work was supported by grants from the National Natural Science Foundation of China (No. 81802278), Huilan Public Welfare-Hansoh Pharma Lung Cancer Precision Medicine Research Fund (No. HL-HS2020-10), and the Natural Science Foundation of Hunan Province (No. 2019JJ50361). The funders had no role in the conceptualization, design, data collection, analysis, decision to publish, or preparation of the manuscript.

## Conflict of Interest

The authors declare that the research was conducted in the absence of any commercial or financial relationships that could be construed as a potential conflict of interest.

## Publisher’s Note

All claims expressed in this article are solely those of the authors and do not necessarily represent those of their affiliated organizations, or those of the publisher, the editors and the reviewers. Any product that may be evaluated in this article, or claim that may be made by its manufacturer, is not guaranteed or endorsed by the publisher.
