# Supplementary material for: A Large Real-World Study on the Effectiveness of the Combined Inhibition of EGFR and MET in EGFR-Mutant Non-Small-Cell Lung Cancer After Development of EGFR-TKI Resistance
Source: Front Oncol. 2021 Oct 1;11:722039. doi: 10.3389/fonc.2021.722039 (PMC8517073; doi:10.3389/fonc.2021.722039)
Supplement: Supplementary file 1 [file Presentation_1.pptx]

## Slide 1
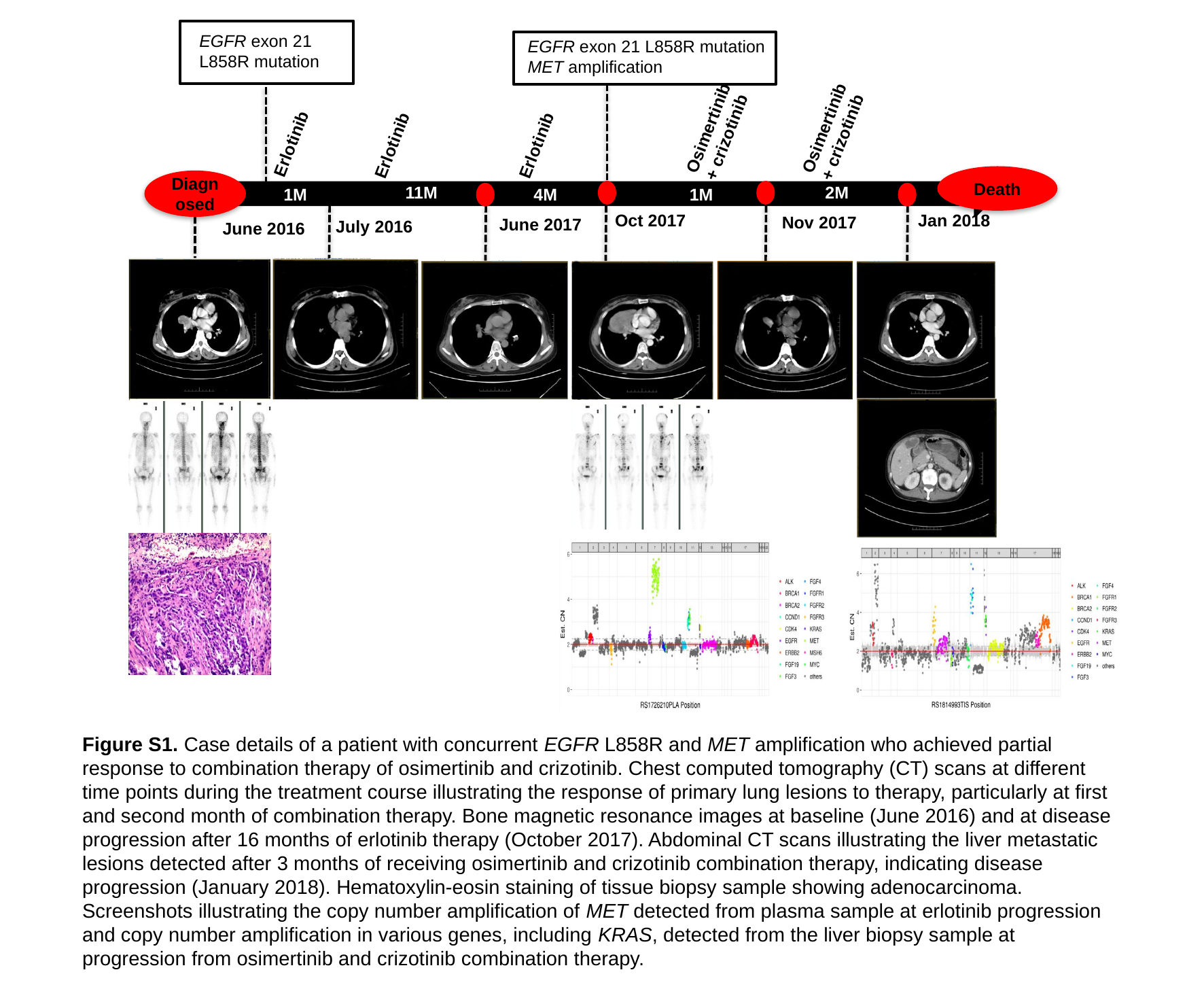

EGFR exon 21 L858R mutation
MET amplification
Osimertinib + crizotinib
Osimertinib + crizotinib
Erlotinib
Erlotinib
Erlotinib
Death
Diagnosed
1M
11M
2M
4M
1M
Oct 2017
Jan 2018
Nov 2017
June 2017
July 2016
June 2016
EGFR exon 21 L858R mutation
Figure S1. Case details of a patient with concurrent EGFR L858R and MET amplification who achieved partial response to combination therapy of osimertinib and crizotinib. Chest computed tomography (CT) scans at different time points during the treatment course illustrating the response of primary lung lesions to therapy, particularly at first and second month of combination therapy. Bone magnetic resonance images at baseline (June 2016) and at disease progression after 16 months of erlotinib therapy (October 2017). Abdominal CT scans illustrating the liver metastatic lesions detected after 3 months of receiving osimertinib and crizotinib combination therapy, indicating disease progression (January 2018). Hematoxylin-eosin staining of tissue biopsy sample showing adenocarcinoma. Screenshots illustrating the copy number amplification of MET detected from plasma sample at erlotinib progression and copy number amplification in various genes, including KRAS, detected from the liver biopsy sample at progression from osimertinib and crizotinib combination therapy.
